# Investigation of Sickle-Cell Haemoglobin Polymerisation under Electrochemical Control

**DOI:** 10.1002/cphc.201300203

**Published:** 2013-05-23

**Authors:** Zeshan Iqbal, Matthew Li, Rachel McKendry, Michael Horton, Daren J Caruana

**Affiliations:** [a] Department of Chemistry, University College London 20 Gordon St., London, WC1 H 0AJ (UK), Fax: (+44) 2076794527 E-mail: D.J.Caruana@ucl.ac.uk; [b] London Centre for Nanotechnology, University College London 17-19 Gordon St, London (UK)

**Keywords:** electrochemistry, haemoglobin, oxygen depletion, protein polymerisation, sickle-cell anemia

## Abstract

We describe an electrochemistry-based technique to control and monitor the polymerisation of sickle-cell haemoglobin (HbS). The polymerisation was monitored as a change in turbidity during the depletion of oxygen in a small volume custom-built thin-layer electrochemical cell. The cell allowed the investigation of HbS polymerisation as a function of HbS concentration, temperature and solution pH. We confirm that the oxygen was efficiently depleted using finite-element modelling to accurately recreate the electrochemical thin-layer cell. Understanding the nucleation and growth of HbS polymerisation will provide a better understanding of the pathophysiology of sickle-cell disease in vivo, and thus help improve therapeutic strategies for this common and frequently disabling disorder.

## 1. Introduction

Sickle-cell disease is a disabling disorder caused by a mutant form of haemoglobin, haemoglobin S (HbS) which polymerises only under hypoxic conditions.[1] HbS differs from normal adult haemoglobin, HbA, by one amino acid on the surface of the ß subunits in which a negatively charged glutamic acid is replaced by a hydrophobic valine residue. This seemingly small change results in the polymerisation of deoxygenated HbS monomers into long insoluble multi-stranded fibres of approximately 21.5 nm diameter.[2, 3] Thus, HbS polymerisation leads to deformation of the red blood cells and occlusion of capillaries, thereby causing haemolytic anaemia, susceptibility to serious infections, stroke, as well as chronic damage to vital organs. Here we present a platform that can be used to investigate the dynamics of polymerisation and potentially be used to identify new therapeutic agents to interrupt the polymerisation.

The kinetics of HbS polymer formation plays a significant role in the pathophysiology of the disease. The double-nucleation mechanism, postulated by Ferrone et al.[4] shows fibre formation and polymerisation is the result of two types of nucleation: homogeneous and heterogeneous. Homogeneous nucleation corresponds to the formation of the rate-determining critical nucleus through thermodynamically unfavourable sequential addition of free HbS monomers, and is characterised by a delay time, during which no polymer is seen. The delay time is followed by an explosive and highly autocatalytic gel formation known as heterogeneous nucleation, which involves nucleation on the surface of pre-existing polymers as well as growth of polymers. The length of the nucleation delay time is strongly dependent on the variables, which alter the solubility of HbS, dependent on factors such as the extent of deoxygenation, HbS concentration, temperature and pH. A simple empirical formula, called the supersaturation equation,[5] relates the delay time, *t*_d_, to the solubility: 1/*t*_d_=*λ*(*c*_o_/*c*_sat_)^*n*^ (*λ* is the proportionality factor and (*c*_o_/*c*_sat_) is the ratio of the initial HbS concentration to the equilibrium solubility). Pathologically, the nucleation delay time is critical as a delay that is longer than the circulation time allows cells to become reoxygenated at the lungs before sickling can occur.

HbS has been studied in great detail and the polymerisation of HbS has probably become the best understood of all protein self-assembly systems.2a However, sickle-cell disease still afflicts millions of people throughout the world, and in particular those from equatorial regions such as sub-Saharan Africa. Previously, we developed a novel analytical technique which monitored HbS polymerisation as a change in turbidity within a thin-layer gold micro-mesh electrochemical cell for potential use as a method that screens blood for sickle-cell anaemia, one that is vastly cheaper and faster than traditional genetic testing.[6] In the present work, a technique to investigate the effect of HbS concentration, temperature, pH and known therapeutic agents on the kinetics and dynamics of gelation at a conducting surface using an microliter volume electrochemical cell to simulate the confinement of a blood vessel.

## 2. Results

**Figure 1:**
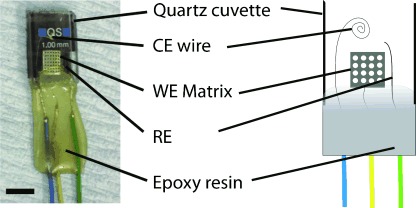
Photograph and diagram of a thin-layer Pt matrix electrochemical cell showing the Pt working electrode (WE matrix) with 350 μm diameter holes drilled in a square-packed array, 100 μm Pt wire counter electrode (CE) and 50 μm Ag wire reference electrode (RE), housed in a 1 mm path length cuvette. Epoxy was used to provide an impervious layer and position the wires. The top of the cuvette was open to the atmosphere. The black line is 5.0 mm scale bar.

The HbS aggregation within the Pt matrix electrochemical thin-layer cell was interrogated using in situ UV/Visible spectroelectrochemistry (Figure 1). The matrix arrangement allowed optical transparency, so the presence of any aggregated protein structures in the apertures, formed due to electrochemical reduction of oxygen in situ, could be detected as a result of wavelength-independent light scattering caused by protein aggregation. The footprint of the light beam covers only four holes in the centre of the Pt matrix. Turbidity values are determined from the absorbance according to the relation *A*=log(*I*_o_/*I*), where turbidity=(*I*_o_/*I*).

Control experiments, performed using HbA instead of HbS for each data set, showed no significant turbidity change at the Pt matrix working electrode, especially at the wavelength range 650 nm to 1100 nm, proving conclusively that any changes in turbidity were due to HbS protein aggregation. The turbidity for all the time traces shown in the following figures is the change in turbidity relative to the starting solution.

**Figure 2:**
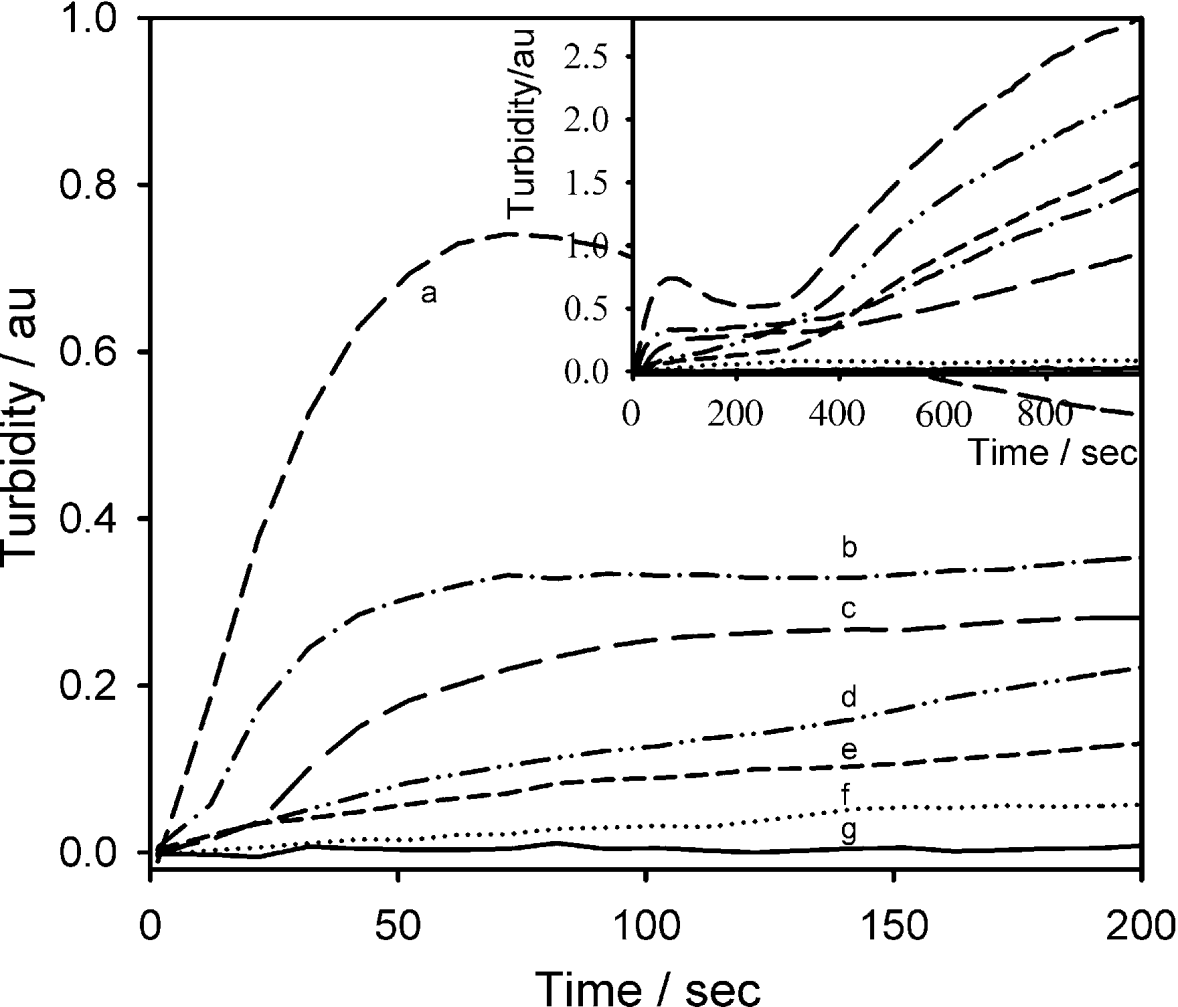
Turbidity–time traces at 700 nm with increasing HbS concentrations between 0 and 200 s, inset showing to 1000 s. Experimental conditions: HbS concentration: a) 100 mg cm^−3^; b) 75 mg cm^−3^; c) 50 mg cm^−3^; d) 40 mg cm^−3^; e) 30 mg cm^−3^; f) 20 mg cm^−3^; g) 100 mg cm^−3^ of HbA. 1.5 m (pH 7.0) phosphate buffer; 0.5 m NaCl; *T*=38 °C; *E*=−0.55 V vs Ag/AgCl.

The effect of increasing HbS protein concentration on turbidity, which is an indication of protein aggregation, at a Pt surface was investigated by performing experiments at protein concentrations between 20 mg cm^−3^ and 100 mg cm^−3^ at 38 °C. Figure 2 shows the effect of protein concentration on the turbidity change at a wavelength of 700 nm (all other wavelengths showed a similar trend), including 100 mg cm^−3^ of HbA as a control experiment.

**Figure 3:**
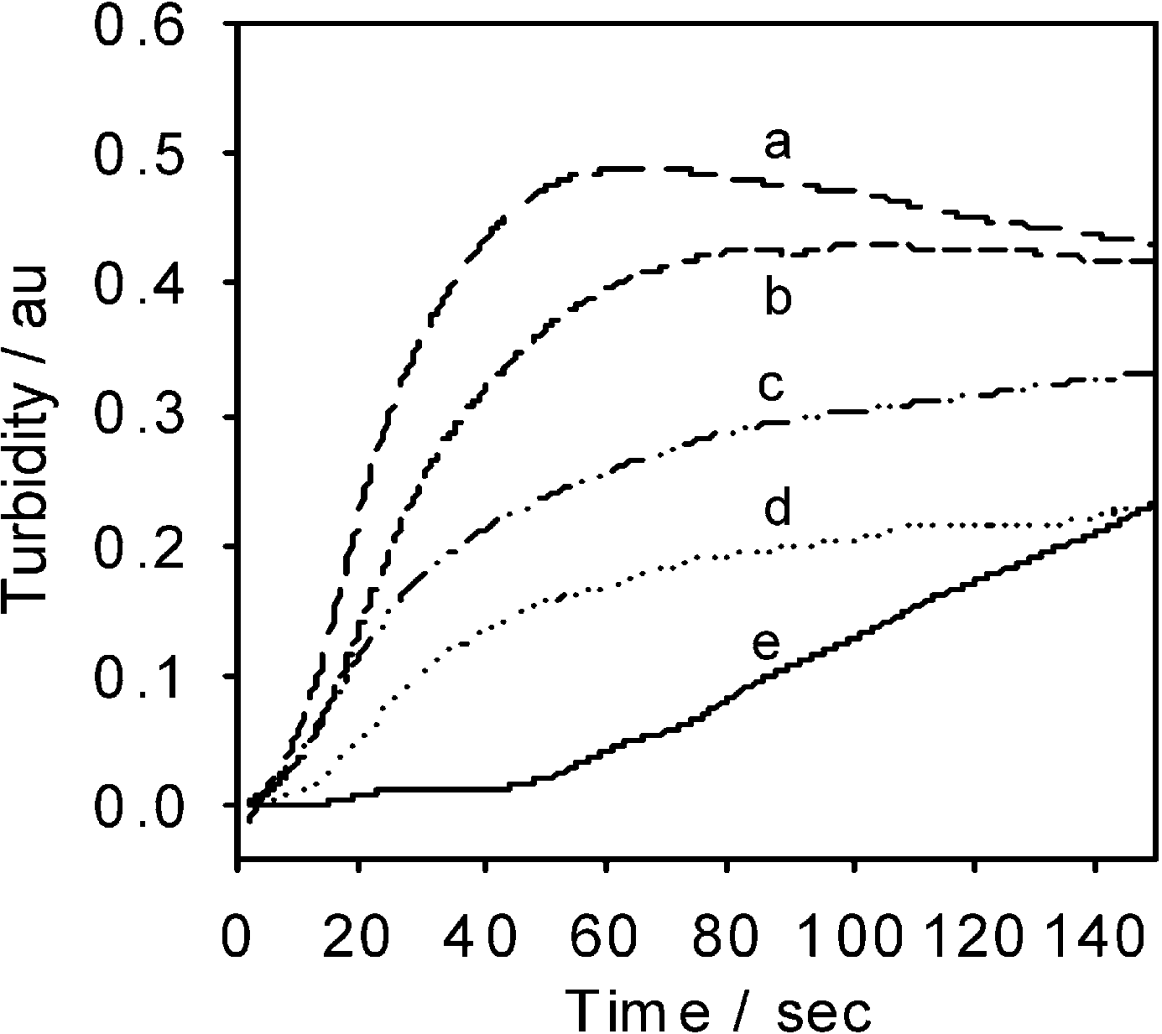
Turbidity–time traces with increasing HbS solution temperature. Conditions: HbS 75 mg cm^−3^; solution temperatures: a) 42 °C; b) 34 °C; c) 38 °C; d) 30 °C; e) 25 °C; 1.5 m (pH 7.0) PBS; 0.5 m NaCl; *E*=−0.55 V vs Ag/AgCl.

The influence of temperature on the HbS fibrillogenesis at the platinum matrix electrode was also investigated, between 25 and 42 °C at 75 mg cm^−3^ HbS concentration, Figure 3. The results in Figure 3 also demonstrated significant HbS aggregation with increasing solution temperature.

**Figure 4:**
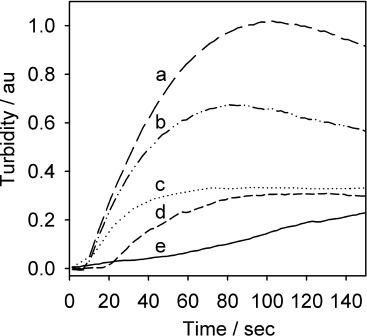
Turbidity–time traces for different buffer pH. Conditions: HbS 75 mg cm^−3^; pH: a) 7.62; b) 7.40; c) 7.20; d) 7.00; e) 6.80; 1.5 m PBS; 0.5 m NaCl; 38 °C; *E*=−0.55 V vs Ag/AgCl.

The effect of changing pH on turbidity was investigated by performing experiments in pH of 6.80, 7.00, 7.20, 7.40 and 7.62 at HbS protein concentrations of 75 mg cm^−3^ (Figure 4). 0.5 m NaCl was used as an additive and the experiments was performed at a temperature of 38 °C. The oxygen-reduction potential changed very slightly (>0.05 V) over this relatively small pH range (0.82), therefore the rate at which the oxygen reduction would probably change is very little, as the potential is held in the diffusion limited region.

**Figure 5:**
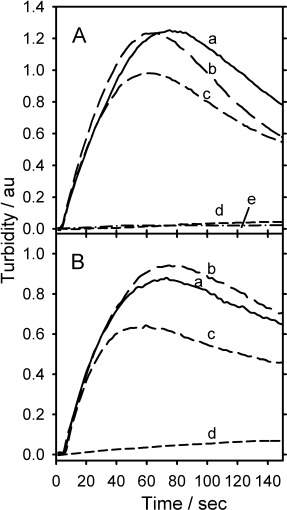
Turbidity–time traces in the presence of A) vanillin and B) 5HMF, chemcial structures shown. Conditions: HbS 100 mg cm^−3^ (1.16 mm); pH 7.00; 1.5 m PBS; 0.5 m NaCl; 38 °C; *E*=−0.55 V vs Ag/AgCl, a) 0 mm; b) 0.58 mm; c) 1.16 mm; d) 11.6 mm for Vanillin and 5HMF respectively, e) in (A) HbA only.

A series of experiments were performed to investigate HbS polymerisation in the presence of two chemical agents known to interrupt the protein polymerisation. Vanillin (2,4-dihydroxybenzaldehyde) and 5-hydroxymethyl-2-furfural (5HMF) were added to the electrochemical cell at different concentrations and essentially the same turbidity time experiment was carried out to measure the protein concentration when the oxygen was depleted. The chemical agent in both cases was incubated in the protein solution 5 min prior to adding the solution to the cell and commencing the experiment. Figures 5 A,B show unambiguously that in the presence of a high concentration of vanillin and 5HTC the extent of polymerisation was reduced dramatically. Cyclic voltammetry in the region of −0.55 V vs Ag/AgCl showed that both vanillin and 5HMF did not have any electrochemical activity.

## 3. Discussion

There is clearly a link between the presence of HbS in the cell and an increase in turbidity with time when the oxygen is consumed by the electrochemical reduction. The square-packed arrangement of holes provided individual tubes within which oxygen was depleted and HbS polymerisation occurred. To ensure that the oxygen has been depleted we recorded the UV/Vis spectra for the haemoglobin before and after oxygen depletion and separately, the oxygen consumption at the matrix electrode was modelled using finite-element software.

**Figure 6:**
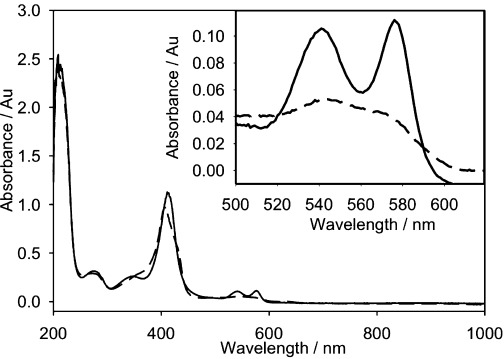
In situ spectroelectrochemistry showing the conversion of oxygenated HbS to deoxygenated HbS at the working electrode, before electrochemical depletion of oxygen (—) and after 1000 s (- - - -). The insert shows the absorbance between 500 to 620 nm for the same spectra. Experimental conditions: HbS concentration 10 mg cm^−3^; 1.5 m pH 7.0 phosphate buffer; 0.5 m NaCl; *E*=−0.55 V vs Ag/AgCl.

Conversion of the oxygenated to deoxygenated state Hb was determined using UV/Vis spectroscopy, through the formation of a characteristic single broad peak at ∼560 nm, as shown in Figure 6. Furthermore, the production of hydrogen peroxide (H_2_O_2_), possible from the electrochemical reduction of oxygen, can cause Hb degradation and haem loss. This was carefully monitored using a spectrophotometric assay to ensure that no H_2_O_2_ was being produced during the experiment. This spectrophotometric assay is described in more detail in a previous paper.[6] There was no measureable hydrogen peroxide produced.

**Figure 7:**
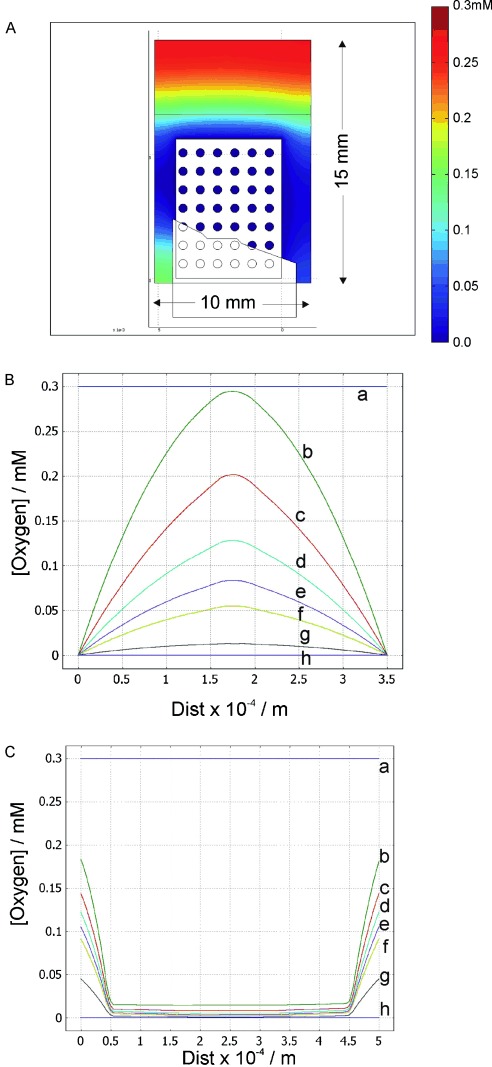
A) Finite-element model of the matrix electrochemical cell using Comsol showing the concentration of O_2_ in and around the Pt matrix electrode at 1000 s. B) Plots of the change in O_2_ concentration horizontally across a single hole. C) Vertically 50 μm away from the electrode and 5 μm into the bulk at either end of the hole at various times. The plots labeled in (B) and (C) are at: a) 0 s; b) 1 s; c) 2 s; d) 3 s; e) 4 s; f) 5 s; g) 10 s; h) 100 s.

The modelling of oxygen depletion at the matrix thin layer electrochemical cell during the experiment was explored by theoretical modelling using Comsol finite-element software. The thin-layer electrochemical cell was recreated to scale, using reference diagrams and photographs of the electrochemical cell. The model was prepared such that different sections of the cell could be explored in detail as shown in Figure 7 A, and was described by nonlinear-diffusion equations in the bulk liquid [Eqs. (1)]:


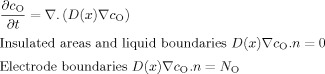
(1)

where *c*_O_, *N*_O_ and *D*(*x*) represent the concentration, outward flux of oxygen and the function of diffusion as a function of *x* respectively. In the case of the electrode boundaries, the flux was approximated by using the Butler–Volmer equation[7] to derive a current, and when the charge of O_2_ was given, could be used to deduce the depletion of oxygen. As oxygen was the reactant of interest (and was being depleted), using an initial oxygen concentration in an air-saturated buffer of 0.3 mm with the diffusion coefficient of oxygen at 3.42×10^−5^ cm^2^ s^−1^, the flux equation could be simplified to Equation (2):


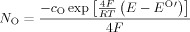
(2)

The model was solved for a range of time spanning the experiment up to 1000 s, the results for these are presented in Figures 7 A–C.

The oxygen concentration before any electroreduction was at a constant level of 0.3 mm (air-saturated buffer) throughout the cell. After 1000 seconds the oxygen concentration of the solution inside and in the near vicinity of the electrode decreased to close to zero (Figure 7 A). For the purposes of this study the area of interest is within the small holes. As clearly seen in Figures 7 B,C, complete depletion of oxygen within the holes of the electrode occurred by 100 s, but significant depletion had occurred after 5 s. Beyond 100 s there was little change in the results. The oxygen concentration moving away from the electrode was still high due to the continued diffusion of atmospheric oxygen into the cell throughout the experiment. Therefore, simulations showed that the liquid that was in close proximity to the electrode experienced and maintained a low oxygen concentration when compared to areas that were not in close contact with the electrode. From modelling oxygen depletion it is clear that the depletion of oxygen is rapid, so it is likely that the limiting factor in fibre formation is not oxygen depletion.

The turbidity time plots for different concentrations of HbS showed two distinct phases, which were clear at protein concentrations greater than 20 mg mL^−1^. The first phase occurred at short times up to 230 s, and then after this a larger increase in turbidity. These two phases can be correlated with the oxygen depletion first within the holes at short times, followed by the slower depletion of oxygen between the platinum matrix electrode and the walls of the cuvette. The increase in turbidity after the reduction phase is due to the depletion of oxygen between the matrix cell and the quartz window which then induces the polymerisation in this area, as shown in Figure 7 C.

Figure 3 demonstrates the increase in turbidity profiles with increasing temperature. The extent of HbS aggregation is increased with warmer protein solutions due to increased kinetic energy of the molecules leading to a greater number of molecular collisions occurring. The maximum turbidity is seen at 34 °C, followed by a decrease and then an increase again at 42 °C. The decrease in turbidity relative to the other temperatures is consistent with the free solution literature, reporting that the minimum solubility of HbS is at 35 °C where the solubility then increases again at higher temperatures.[8] The difference between the free solution experiment and the conducting surface experiments could be due to differences in the mechanism of protein aggregation at a liquid–liquid interface as compared to liquid–solid interfaces.

To obtain kinetic information we can analyse the data using the kinetic scheme developed for the fibrillation mechanism of human calcitonin by Kamihira et al.[9] and subsequently modified for β-amyloid fibrillogenesis by Sabaté et al.[10] and Iqbal et al.[6] for HbS, which was fitted to our data. The kinetics of the process is controlled by two key parameters, namely the nucleation rate *k*_1_ [Eq. (3)]:


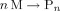
(3)

and the growth described by the rate constant *k*_2_ [Eq. (4)]:


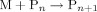
(4)

with M representing monomeric HbS, *n* the number of HbS molecules and P_*n*_ the fibre nucleus. The overall kinetic equation for the nucleation and growth of the fibres can be expressed by Equation (5):


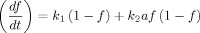
(5)

where *f* is the fraction of fibres in the system and *a* is the initial concentration of HbS in the solution. Equation (5) can be integrated under the boundary condition of *t*=0, *f*=0, to give Equation (6):


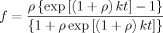
(6)

where *k*=*k*_2_*a* and *ρ*=*k*_1_/*k*. We have used least-squares fitting of Equation (6) to the turbidity data at 800 nm for the first 120 seconds to obtain values of *k*_1_ and *k*_2_.

The *k*_1_ and *k*_2_ values for different temperatures were used to calculate activation energies for the nucleation and elongation process using typical Arrhenius analysis. The nucleation activation energy, and elongation activation energy were calculated to be 124.63±13.42 kJ mol^−1^ and 99.03±15.61 kJ mol^−1^ respectively. The activation energy for elongation is similar to that determined for β-amyloid fibrillogenesis, (95.30±4.60 kJ mol^−1^).[10] However, the elongation activation energy, defined as the energy required for the initiation of the elongation phase, was lower than expected (311.20 kJ mol^−1^ for β-amyloid). The nucleation phase is normally the rate-determining step and would be larger in magnitude. This may be partly explained due to the fact that in our system nucleation occurred at a surface rather than free in solution, which is thermodynamically more favourable.

The aggregation of HbS was shown to be markedly dependent on the pH as the results obtained (Figure 4) show that protein aggregation is favoured by a slightly alkaline pH, with the extent of polymer growth being greatest at pH of 7.62 and 7.40. At a pH of 7.40 and 7.62 monomeric HbS carries approximately one negative charge as the isoelectric point of molecular HbA is 6.80.[11] However, the overall net charge of the polymeric protein is not known as the effect of a structural change into an ordered gel-like system of HbS polymers on the pI value has not been established. Therefore, the high level of turbidity seen at pH 7.62, as compared to other pHs, could possibly have been due to the stabilisation of HbS aggregates through a decrease in the net electrostatic repulsion between the polymers. Alternatively, a more basic pH could have provided greater hydrophobic specific interactions between HbS monomers, leading to enhanced polymerisation of HbS fibres.

Previous free solution studies have shown that aggregation of HbS is markedly dependent on the pH.[12] These studies have shown that the solubility changes very little between pH 6 and 7, but increases sharply at more acid and alkaline values, probably because of changes in the ionisation state of histidines. Furthermore, these studies have indicated that the minimum in the solubility–pH profile is at pH 6.5. In this experiment, polymerisation at a negatively charged surface was favoured by an increase in buffer pH. Therefore, the presence of a negatively charged conducting surface may have an impact on not only the polymer–polymer interactions but also polymer–electrode interactions.

Consequently, the results obtained from this particular pH study disagree with the literature reported pH value of 6.50 as a direct consequence of the negative charge biased surface. The presence of a conducting surface carrying negative charge has a major effect on not only the polymer–polymer interaction but also polymer–electrode interaction.

In the presence of polymerisation disruption agents the effect was very similar for both vanillin and 5HMF. Up to 1.16 mm of Vanillin or 5HMF showed similar significant polymerisation. Increasing the concentration tenfold to 11.6 mm shows a marked decrease in polymerisation to the point that it was barely measureable. This concentration corresponded to ten times the concentration of HbS present in the solution. Vanillin (Figure 5 A) decreases HbS polymerisation by allosterically modulating the HbS molecule to the high affinity state, and concurrently inhibiting the T state HbS polymerisation. The mode of action of 5HMF is very similar to that of vanillin—it acts as an allosteric affector to shift the affinity of oxygen. From Figure 5, both vanillin and 5HMF show similar effectiveness as a polymerisation inhibitor.[13]

Both these chemical agents are naturally occurring. Vanillin is an extremely good candidate as an anti-sickling agent as it is a food additive on the GRAS (generally regarded as safe) list and has little adverse effects at high dosages, around 1 to 4 gm d^−1^ or less.[14] However, vanillin has low oral bioavailability due to rapid decomposition in the digestive tract, and thus a pro-drug of vanillin has been developed. The pro-drug, MX-1520, has been investigated in rodents and shown to be bioavailable after oral administration in rats and still to retain its efficacy.[14, 15] 5HMF was found to be rapidly absorbed into the bloodstream, binding and modifying HbS molecules at levels as high as 90 % without being destroyed in the gastrointestinal tract.[13]

## 4. Conclusions

We describe an optically transparent electrochemical thin-layer cell to investigate the polymerisation of HbS at a conducting surface. The study shows that the design of this particular cell using finite-element modelling of oxygen depletion in the matrix cell was reduced within 20 s of the start of the experiment. Experiments performed with varying protein concentration showed a large concentration dependence on the HbS fibre aggregation. It was also shown that aggregation of HbS at a conducting Pt matrix surface was dependent on the temperature in the range 25 °C to 42 °C and that polymer growth at a conducting surface was favoured by elevated temperatures, although the solubility of the protein showed a decrease at 34 °C and 42 °C. This is in reasonable agreement with literature. The differences may be due to the mechanism of protein aggregation in a homogenous liquid system as opposed to at a solid interface as in our case. Moreover, the investigations revealed that protein aggregation was favoured by a slightly alkaline pH with the extent of polymer formation being greatest at pH levels of 7.62 and 7.40.

This study has shown the possibility of using this electrochemical matrix cell as a screening device even when there is limited availability of protein, whilst also showing the great importance of coupling concentration with temperature to achieve an accelerated fibre growth rate for future use as a screening device. A screening device could be used to test a variety of compounds which disrupt the polymer formation as a route to a therapeutic strategy for this disease. Knowledge of how these parameters affect the kinetics and dynamics of nucleation and growth of HbS polymerisation at a surface will provide a better understanding of the pathophysiology of sickle-cell disease in vivo in order to improve therapeutic strategies for this common, and frequently disabling, genetic disorder.

## Experimental Section

### Materials and Instrumentation

De-ionised water (Millipore Milli-Q gradient, <0.05 S cm^−2^) was used for all solutions. HbS, HbA, vanillin (2,4-dihydroxybenzaldehyde) and 5-hydroxymethyl-2-furfural (5HMF) and other chemicals were purchased from Sigma chemical company (Poole, UK) and used as supplied. Unless otherwise stated the supporting electrolyte was 1.5 m (pH 7.0) phosphate buffer solution containing 0.5 m sodium chloride.

Chronoamperometry was carried out in a conventional three-electrode system comprising a counter, working and reference electrodes connected to a potentiostat (μAutolab Type II, Eco Chemie B.V. supplied by Windsor Scientific Ltd, UK), controlled by GPES software (version 4.9, Eco Chemie B. V. Utrecht, Netherlands). In the three-electrode system, consisting of a Pt sheet (thickness 0.35 mm, 3 mm×4 mm, 99.99+ % purity, Advent Materials), into which regular holes of diameter 350 μm were drilled (referred to as the Pt matrix), was used as the working electrode, a Pt coil (diameter 100 μm, Advent Materials) served as the auxiliary electrode whilst all potentials were given versus an oxidised silver wire quasi-reference electrode (Ag/AgCl). These electrodes were arranged in a modified quartz cuvette (1 mm path length, Hellma, UK) as shown in Figure 1. The cell had a typical total volume of 100 μL±5 μL. For the spectroscopy experiments, the entire cuvette, except for a small window on the Pt matrix working electrode (1.0 mm×1.5 mm), was blanked for the light beam. All materials used in the fabrication of the cell were thoroughly cleaned prior to its construction using acetone.

Ultraviolet and visible absorption spectra were recorded with an Agilent 8453 UV/Vis spectrophotometer in kinetic mode on a quartz cuvette. Spectra were recorded using UV/Vis ChemStation software (Rev. A.09.01). The temperature was maintained and controlled with the use of a 2.5×2.5 cm Peltier device (Thermo Electric Cooler Type DT1069; Marlow Industries Inc., USA).

### Procedure

The electrodes were electrochemically cleaned by cycling in 0.1 m sulphuric acid until there was no further change in the voltammetric response. 100 μL of the Hb solution was transferred into the cuvette and capillary action ensured that all the electrodes were immersed in the solution. The cuvette was fixed on to the thermostat to ensure the required solution temperature was achieved and placed in the spectrophotometer, in such a way that both the spectrophotometer and the potentiostat could be operated in tandem. In situ deoxygenation was performed by electrochemical reduction at the electrode (*E*=−0.55 V vs the quasi-reference electrode) as well as absorbance measurements for up to 1000 s with spectra every 2 s or 10 s intervals. The wavelength acquisition range was from 200–1100 nm and time traces at 600, 650, 700 and 800 nm were monitored.
